# Genetic Characteristics of the Coxsackievirus A24 Variant Causing Outbreaks of Acute Hemorrhagic Conjunctivitis in Jiangsu, China, 2010

**DOI:** 10.1371/journal.pone.0086883

**Published:** 2014-01-24

**Authors:** Bin Wu, Xian Qi, Ke Xu, Hong Ji, Yefei Zhu, Fenyang Tang, Minghao Zhou

**Affiliations:** Department of Acute Infectious Disease Control and Prevention, Jiangsu Province Center for Disease Control and Prevention, Nanjing, Jiangsu, China; University of Texas Medical Branch, United States of America

## Abstract

During September 2010, an outbreak of acute hemorrhagic conjunctivitis reemerged in Jiangsu, three years after the nationwide epidemic in China in 2007. In total, 2409 cases were reported, 2118 of which were reported in September; 79.8% of those affected were students or teachers, with a median age of 16 years. To identify and demonstrate the genetic characteristics of the etiological agent, 52 conjunctival swabs were randomly collected from four different cities. After detection and isolation, 43 patients were positive for coxsackievirus A24 variant according to PCR and 20 according to culture isolation. Neither adenovirus nor EV70 was detected. A phylogenetic study of the complete 3Cpro and VP1 regions showed that the Jiangsu isolates clustered into a new lineage, GIV-C5, with two uniform amino-acid mutations that distinguished them from all previous strains. Another new cluster, GIV-C4, formed by Indian isolates from 2007 and Brazilian isolates from 2009, was also identified in this study. Interestingly, our isolates shared greatest homology with the GIV-C4 strains, not with the isolates that were responsible for the nationwide acute hemorrhagic conjunctivitis epidemic in China in 2007. Although all our isolates were closely related, they could be differentiated into two subclusters within GIV-C5. In conclusion, our study suggests that a new cluster of coxsackievirus A24 variant that had already evolved into diverse strains was associated with the acute hemorrhagic conjunctivitis outbreaks in Jiangsu in September 2010. These viruses might have originated from the virus isolated in India in 2007, rather than from the epidemic strains isolated in China in 2007.

## Introduction

Acute hemorrhagic conjunctivitis (AHC), characterized by the sudden onset of ocular pain, swelling of the eyelids, a “foreign body” sensation or irritation, epiphora (excessive tearing), eye discharge, and photophobia, is mainly caused by enterovirus 70 (EV70), coxsackievirus A24 variant (CA24v), or adenoviruses [1]–[3]. In China, the first AHC outbreak was reported in 1971 [4]. Because AHC is a notifiable infectious disease in China, all cases diagnosed by physicians have been registered in the National Disease Supervision Information Management System (NDSIMS). In 2007, a nationwide AHC epidemic caused by CA24v was reported in China, with a total of 74,263 AHC cases [5], [6], after which the number of AHC cases returned to baseline in 2008 and 2009. In 2010, outbreaks of AHC reemerged around the world [7]–[11]. In China, outbreaks were reported in Zhejiang, Guangdong, Guangxi, Shandong, Henan, Fujian, Beijing, and Chongqing [7], [12]. Several studies demonstrated that the outbreaks were caused by CA24v. However, to the best of our knowledge, in China in2010, the complete sequences of the VP1 and 3Cpro regions of CA24v, which are usually used for phylogenetic analyses, were only determined in Zhejiang and Guangdong Provinces and submitted to GenBank. These sequences indicated that the CA24v strains causing these outbreaks belonged to Group IV [7], [8]. The objectives of the present study were to investigate the AHC outbreak in Jiangsu Province, China, in September 2010 and to identify the etiological agent causing this outbreak and determine its genetic characteristics.

## Results

### The Outbreak

**Figure 1:**
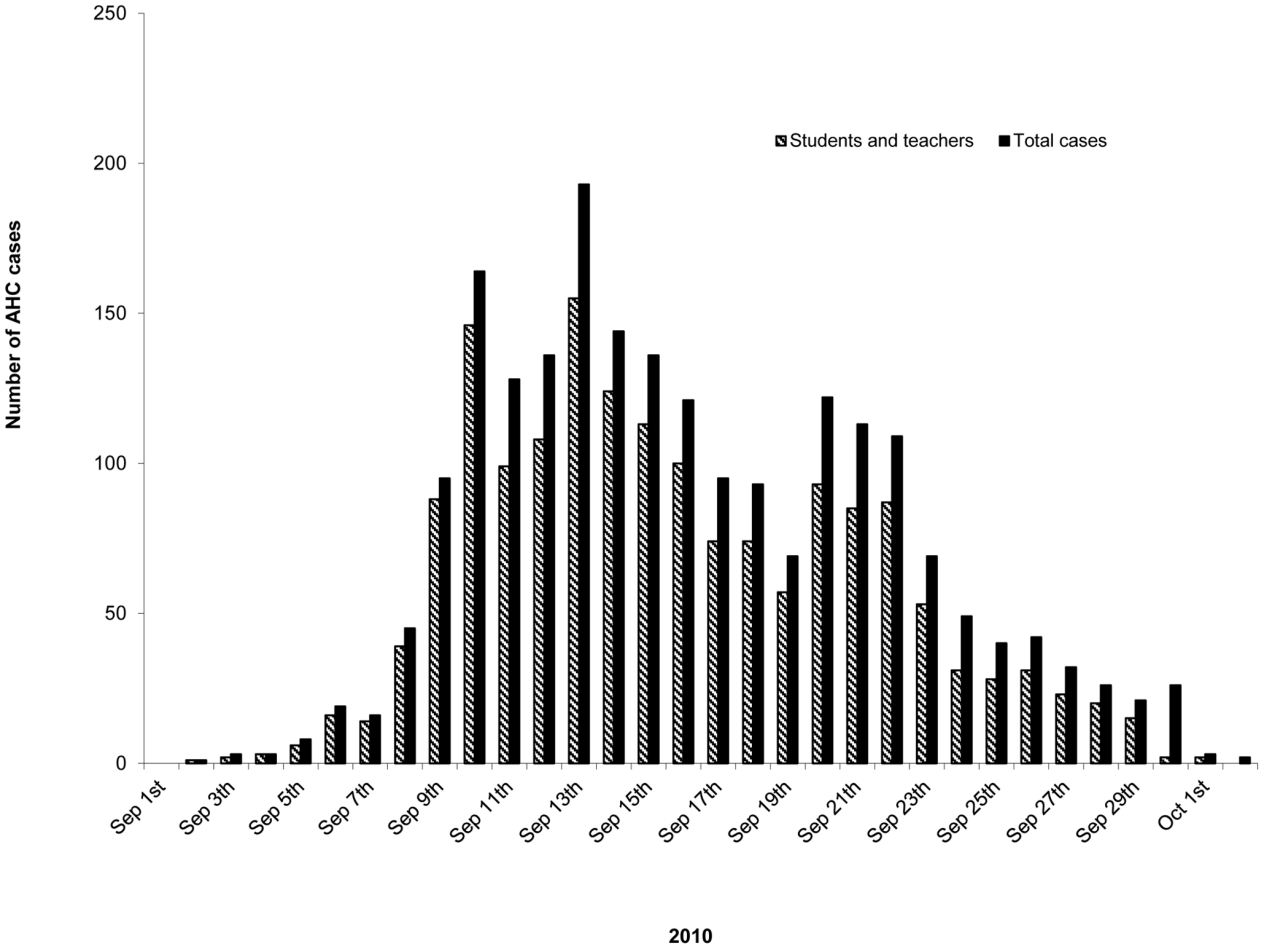
Distribution of the reported AHC cases in Jiangsu Province during the outbreak of September 2010.

Of the 4276 cases of AHC in Jiangsu Province reported to the NDSIMS between January 1 2007 and December 31 2010, 2409 were officially reported in 2010, which was approximately 25-fold higher than in 2009 and 20-fold higher than in 2008, and even 0.5-fold higher than in 2007 (**Table 1**). Unlike previous years, approximately 88% of the patients in 2010 were reported during September in 13 cities of Jiangsu Province. Of all the patients, 79.8% were students, kindergarten children, or teachers (**Table 1**). Reports began to increase from September 5, reached a peak on September 13, and returned to baseline on October 1 (**Fig. 1**). All the patients had conjunctival congestion and a clear history of contact with people presenting similar symptoms. No death from AHC was reported.

**Table 1:** AHC cases reported to NDSIMS in Jiangsu from 2007 to 2010.

Year	Total cases	Cases in September	Student cases	Median age	Male:Female
**2007**	1649	984	702	25 years	1.99∶1
**2008**	122	31	38	30 years	1.44∶1
**2009**	96	8	20	27 years	2.10∶1
**2010**	2409	2118	1690	16 years	1.69∶1

### Virus Detection and Isolation

Neither adenovirus nor EV70 was detected in 52 patients with acute conjunctivitis.

However, 82.7% (43 of 52) of the patients were positive for CA24v according to PCR and 38.5% (20 of 52) according to culture isolation, suggesting that CA24v was the causative agent of the outbreak.

### Phylogenetic Analysis of the 3Cpro Region

**Figure 2:**
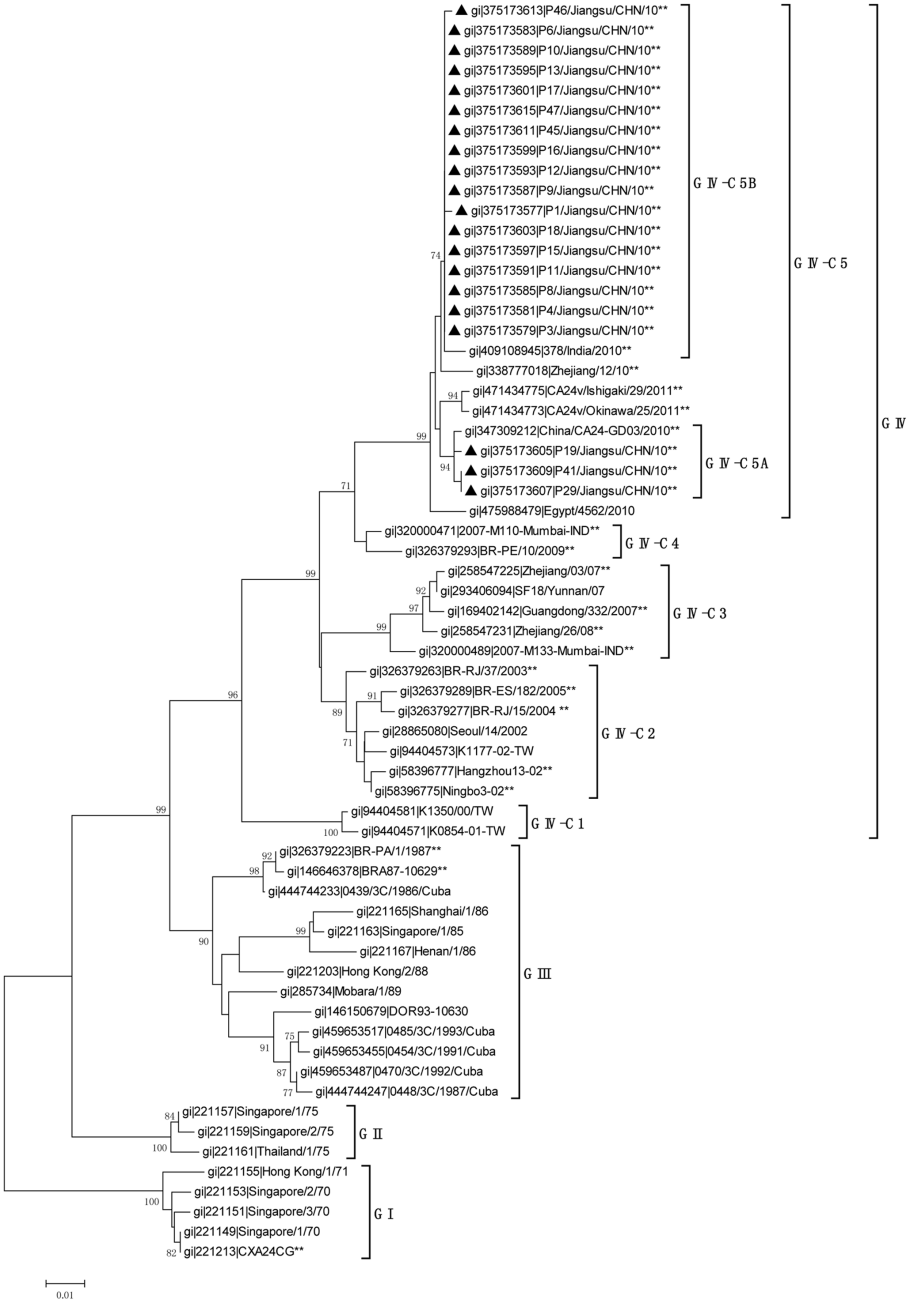
Phylogenetic analysis based on the 549-nucleotide 3Cpro gene of CA24v. All Jiangsu CA24v isolates identified in this study, marked with black triangles, were compared with strains available in GenBank. Strains for which the complete sequences of both the 3Cpro and VP1 regions were available are indicated with **. The MEGA 4.0 software was used for the phylogenetic analysis. The stability of the nodes was assessed using neighbor-joining cluster analysis with 1000 bootstrap replications, and only bootstrap values >70% are shown at the nodes.

As in previous studies [8], [13], phylogenetic analysis of the 3Cpro region showed that all the CA24v strains could be divided into four major groups. Our strains belonged to Group IV and formed a new cluster, C5, with strains isolated throughout the world after 2010. Another new cluster (C4) formed by strains from India (2007) and Brazil (2009) was also identified in this study (**Fig. 2**). Analysis of the sequence identities of all five clusters of Group IV (**Table 2**) showed that our isolates shared the greatest homology with the C4 strains, rather than with the C3 strains, which were responsible for the nationwide AHC epidemic in China in 2007.

**Table 2:** Homology of the complete 3Cpro regions of the strains in different clusters within CA24v genotype GIV investigated in this study.

Homology (%)	GIV-C5	GIV-C4	GIV-C3	GIV-C2	GIV-C1
**GIV-C5**	98.0–100.0	–	–	–	–
**GIV-C4**	95.8–97.0	98.7	–	–	–
**GIV-C3**	92.8–94.2	95.8–96.8	98.0–99.8	–	–
**GIV-C2**	93.8–96.8	95.4–97.0	94.6–96.4	98.0–99.6	–
**GIV-C1**	91.0–91.9	92.5–93.4	91.9–93.6	93.0–94.2	99.5

Further analyses showed that our strains clustered in two subclusters: C5A and C5B (**Fig. 2**). C5A, consisting of isolates P19, P29, and P41, with sequence identities of 99.6%–100%, shared greatest homology with strain GD03/2010 (99.6%). C5B, formed by other strains sharing 99.3%–100% homology, clustered with strain 378/India/2010 (99.3%–99.5%). The homology between our strains and Zhejiang strains isolated in 2010 ranks second to the Indian strain (98.9%–99.1%), which is identical to the homology between C5A and C5B (98.9%–99.1%).

### Phylogenetic Analyses of the VP1 Region

**Figure 3:**
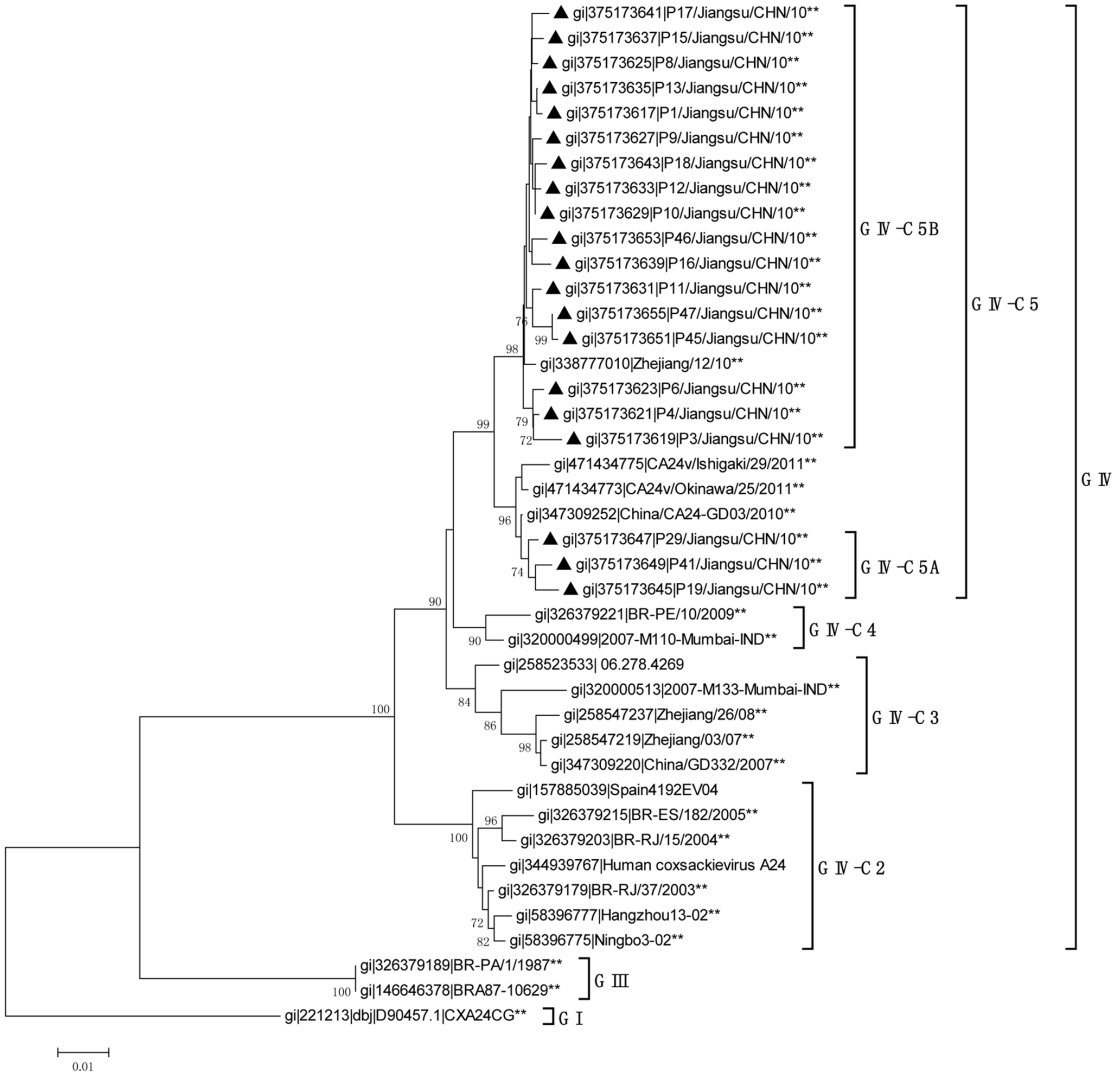
Phylogenetic analysis based on the 915-nucleotide VP1 gene of CA24v. All Jiangsu CA24v isolates identified in this study, marked with black triangles, were compared with strains available in GenBank. Strains for which the complete sequences of both the 3Cpro and VP1 regions were available are indicated **. The MEGA 4.0 software was used for the phylogenetic analysis. The stability of the nodes was assessed using neighbor-joining cluster analysis with 1000 bootstrap replications, and only bootstrap values >70% are shown at the nodes.

The sequence homology of the VP1 region confirmed the findings of the 3Cpro region analysis (**Fig. 3** and **Table 3**). The C5 lineage, which included our strains, also shared the highest sequence identities with the C4 lineage, which contained the same strains as in the 3C region analysis. The subclusters C5A and C5B were similarly identified with the VP1 region. The sequence identity between C5A and C5B ranged from 97.4% to 98.3%. As before, C5A shared greatest homology with strain GD03/2010 (99.2%–99.7%). However, because there were no complete VP1 sequences for the Indian strains isolated in 2010, we demonstrated that C5B clustered and shared its greatest homology with strain Zhejiang/12/10 (99.0–99.6%).

**Table 3:** Homology of the complete VP1 regions of the strains in different clusters within CA24v genotype GIV in this study.

Homology (%)	GIV-C5	GIV-C4	GIV-C3	GIV-C2
**GIV-C5**	97.3–99.9	–	–	–
**GIV-C4**	96.5–97.7	98.9	–	–
**GIV-C3**	94.1–97.3	96.1–97.5	97.4–99.8	–
**GIV-C2**	94.1–95.4	94.5–95.8	94.5–96.3	98.0–99.6

### Amino Acid Sequence Analyses of the 3Cpro and VP1 Regions

An amino acid sequence analysis revealed two uniform mutations in all the strains that formed the C5 lineage after 2010. One was the *isoleucine* at position 54 of the 3Cpro region, and the other was the *aspartic acid* at position 301 of the VP1 region, whereas these two positions contained *valine* and *asparagine*, respectively, in all the other strains available in the GenBank before 2010 (**Table 4** and **Table 5**).

**Table 4:** Amino acid mutations in the 3Cpro region in different genotypes of CA24v.

Genotype	Strains	Amino acid site in the 3Cpro region																	
		8	15	31	47	49	54	68	76	78	92	114	118	139	151	154	158	160	182
**GI**	gi|221213|CXA24CG	V	I	H	I	I	V	T	K	K	T	V	A	N	I	T	I	M	S
	gi|221149|Singapore/1/70	.	.	.	.	.	.	.	.	.	.	.	.	.	.	.	.	.	.
**GII**	gi|221161|Thailand/1/75	.	.	.	V	.	.	.	.	.	.	.	.	H	.	V	.	.	N
	gi|221159|Singapore/2/75	.	.	.	.	.	.	.	.	.	.	.	.	H	.	V	M	.	N
**GIII**	gi|221167|Henan/1/86	.	V	Y	.	.	.	.	.	.	A	.	T	H	.	.	.	.	.
	gi|146646378|BRA87-10629	I	V	.	.	.	.	.	R	.	A	.	.	H	.	.	.	.	.
**GIV-C1**	gi|94404581|K1350/00/TW	.	V	.	.	.	.	I	.	.	.	.	.	H	.	.	.	.	.
	gi|94404571|K0854-01-TW	.	V	.	.	.	.	I	.	.	.	.	.	H	.	.	.	.	.
**GIV-C2**	gi|58396777|Hangzhou13-02	.	V	.	.	.	.	I	.	.	.	I	.	H	V	.	.	I	.
	gi|58396775|Ningbo3-02	.	V	.	.	.	.	I	.	.	.	I	.	H	V	.	.	I	.
	gi|326379277|BR-RJ/15/2004	.	V	.	.	.	.	I	.	.	.	I	.	H	V	.	.	I	.
**GIV-C3**	gi|320000489|2007-M133-IND	.	V	.	.	V	.	V	.	.	.	I	.	H	.	.	.	I	.
	gi|169402142|Guangdong/332/2007	.	V	Y	.	.	.	V	.	.	.	I	.	H	.	.	.	I	.
	gi|258547231|Zhejiang/26/08	.	V	Y	.	.	.	V	.	.	.	I	.	H	.	.	.	I	.
**GIV-C4**	gi|320000471|2007-M110-IND	.	V	.	.	.	.	.	.	.	.	I	.	H	V	.	.	I	.
	gi|326379293|BR-PE/10/2009	.	V	.	.	.	.	.	.	.	.	I	.	H	V	.	.	I	.
**GIV-C5**	gi|475988479|Egypt/4562/2010	.	V	.	.	.	I*	.	.	.	.	I	.	H	V	.	.	I	.
	gi|409108945|378/India/2010	.	.	.	.	.	I*	.	.	.	.	I	.	H	V	.	.	I	.
	gi|375173607|P29/Jiangsu/CHN/10	.	V	.	.	.	I*	.	.	.	.	I	.	H	V	.	.	I	.
	gi|347309212|GD03/2010	.	V	.	.	.	I*	.	.	.	.	I	.	H	V	.	.	I	.
	gi|338777018|Zhejiang/12/10	.	V	.	.	.	I*	.	.	R	.	I	.	H	V	.	.	I	.
	gi|471434775|Ishigaki/29/2011	.	V	.	.	.	I*	.	.	.	.	I	.	H	V	.	.	I	.

* Amino acid mutation only present in all GIV-C5 strains.

**Table 5:** Amino acid mutations in the VP1 region in different genotypes of CA24v.

Genotype	Strains	Amino acid site in the VP1 region																
		11	25	32	51	56	89	100	103	146	151	168	196	250	255	256	297	301
**GI**	gi|221213|CXA24CG	S	L	S	V	I	M	E	K	T	Y	R	I	F	I	I	I	N
**GII**	gi|146646378|BRA87-10629	T	S	P	A	V	I	.	.	A	H	.	M	Y	.	T	T	.
**GIV-C2**	gi|58396775|Ningbo3-02	T	P	L	A	V	I	D	R	A	H	.	M	Y	.	T	T	.
	gi|58396777|Hangzhou13-02	T	P	L	A	V	I	D	R	A	H	.	M	Y	V	T	T	.
	gi|326379179|BR-RJ/37/2003	T	P	L	A	V	I	D	R	A	H	.	M	Y	.	T	T	.
	gi|157885039|Spain4192EV04	T	P	L	A	V	I	D	R	A	H	.	M	Y	.	T	T	.
	gi|326379209|BR-ES/93/2005	T	P	L	A	V	I	D	R	A	H	.	M	Y	.	T	T	.
**GIV-C3**	gi|347309220|GD332/2007	T	H	L	A	V	I	D	R	A	H	.	M	Y	.	T	T	.
	gi|320000513|2007-M133-IND	T	H	L	A	V	I	D	R	A	H	.	M	Y	.	T	T	.
	gi|258547237|Zhejiang/26/08	T	H	L	A	V	I	D	R	A	H	Q	M	Y	.	T	T	.
**GIV-C4**	gi|320000499|2007-M110-IND	T	H	L	A	V	I	D	R	A	H	.	M	Y	.	T	T	.
	gi|326379219|BR-PE/9/2009	T	H	L	A	V	I	D	R	A	H	.	M	Y	.	T	T	.
**GIV-C5**	gi|338777010|Zhejiang/12/10	T	H	L	A	V	I	D	R	A	H	.	M	Y	.	T	T	D*
	gi|347309252|CA24-GD03/2010	T	H	L	A	V	I	D	R	A	H	.	M	Y	.	T	T	D*
	gi|375173647|P29/Jiangsu/10	T	H	L	A	V	I	D	R	A	H	.	M	Y	.	T	T	D*
	gi|471434775|Ishigaki/29/2011	T	H	.	A	V	I	D	R	A	H	.	M	Y	.	T	T	D*

* Amino acid mutation only present in all GIV-C5 strains.

## Discussion

Our study demonstrates that CA24v was the causative agent of the AHC outbreak in Jiangsu Province in September 2010. CA24v is a major etiological agent of AHC. After its first description in Singapore in 1970, AHC outbreaks caused by CA24v have occurred periodically throughout the world [2], [4], [8]–[11], [13]–[17]. According to previous studies, four genotypes of CA24v can be distinguished and identified by phylogenetic analysis of the 3Cpro and VP1 regions [18], [19]. Pei-Yu Chua reported that the CA24v strains of the fourth genotype, GIV, isolated from 2000 to 2007, can be divided into three different clusters: GIV-C1, GIV-C2, and GIV-C3 [13]. The Chinese isolates isolated in Guangdong, Yunnan, and Zhejiang provinces in 2007 and 2008 were all clustered within GIV-C3 [5]–[6], [8]. In this study, two new clusters were identified containing strains isolated after 2007. Our strains belonged to GIV-C5, and the strains from India (2007) and Brazil (2009) formed GIV-C4 (**Fig. 2** and **Fig. 3**). Contrary to previous studies [7], [8], the differentiation of GIV-C5 and GIV-C4 is supported by phylogenetic analyses. First, our strains shared the highest sequence identities with the other strains within their clusters, whereas the identities were much lower with isolates that were categorized outside their clusters. The sequence identities were similar to the scores for the strains of other clusters (C1, C2, and C3) when compared with intra- and extra-clusters (**Table 2** and **Table 3**). Second, amino acid analyses revealed two uniform mutations that were only present in all the strains that formed the C5 lineage (**Table 4** and **Table 5**). These results suggest that the CA24v strain that caused the AHC outbreak in Jiangsu in September 2010 belonged to the new C5 cluster.

Interestingly, the new C5 cluster shared greatest homology and clustered with the C4 strains, which were represented by Indian strain M110 isolated in 2007 and Brazilian strain BR-PE/10 isolated in 2009 [10], [15], and not with C3, which was responsible for the nationwide AHC epidemic in China in 2007. However, other Indian strains, such as M133 of GIV-C3, were also isolated in 2007. These results suggest that clues to the differentiation of C3 and C4 can be found retroactively as early as 2007 in India. C5 might then have evolved from C4, and has since evolved differently from the Chinese isolates of 2007 (**Fig. 2** and **Fig. 3**).

Our isolates were also differentiated as two subclusters within C5. The C5A cluster, formed by P19, P29, and P41, grouped together with the strains isolated in Guangdong Province in China in 2010 [8], whereas the others that formed C5B clustered with the strains isolated in Zhejiang Province in China in 2010. In an analysis of all the strains available in GenBank, we found that no strain isolated in 2010 in Guangdong clustered with C5B and no strain isolated in the same year in Zhejiang clustered with C5A. In other words, in 2010, at least when and where the studies were performed, the CA24v outbreaks in Guangdong might have been caused by strains that clustered with C5A alone, whereas in Zhejiang, CA24v only clustered with C5B [7], [8]. However, in Jiangsu, the two subclusters were epidemic at the same time.

C5A shared the greatest sequence identity in both the 3Cpro and VP1 regions with strains from Guangdong Province in 2010 [8]. The VP1 region of C5B also shared the greatest identity with the strains isolated in Zhejiang Province in 2010 [7]. However, the 3Cpro region of C5B shared its highest homology with the strain isolated in India in the same year. Although the complete VP1 region of the Indian isolate was not available in GenBank, we deduced that frequent mutations and/or recombination events have occurred within the C5 cluster, generating its diversity.

In conclusion, our study suggests that a new cluster of coxsackievirus A24 variant, which had already evolved into diverse strains, was associated with the acute hemorrhagic conjunctivitis outbreak in Jiangsu in September 2010. These viruses might have originated from the Indian strains isolated in 2007, rather than from the epidemic strains present in China in 2007.

## Materials and Methods

### Ethics Statement

This study was approved by the Ethics Committee of the Jiangsu Provincial Center for Disease Control and Prevention. Written informed consent for the use of the 52 clinical specimens for virus isolation and detection was obtained from all the patients or the legal guardians of child patients. The Ethics Committee waived the requirement for the consent of the patients or guardians to use the basic information about the 4276 cases of AHC obtained from NDSIMS, because no samples were acquired from those patients. Patient identifiers, including names, addresses, and case numbers, were removed from any parts of the documents used in this study to ensure patient confidentiality.

### Clinical Information and Specimen Collection

The 4276 cases of AHC in Jiangsu Province that were reported to NDSIMS between January 1 2007 and December 31 2010 were investigated in this study. In total, 52 conjunctival swabs from AHC patients aged 7–56 years (28 males and 24 females in the acute phase), were randomly collected from four hospitals in different cities (13 from Nanjing, eight from Taizhou, 15 from Yancheng, and 16 from Lianyungang) during September 2010 (1–5 days after the onset of symptoms).

### Virus Detection and Isolation

Viral nucleic acid was extracted from the swabs with the QIAamp Mini Viral RNA Extraction Kit and the DNeasy Blood & Tissue Kit (Qiagen, Hilden, Germany). RT–PCR was performed with primers specific for CA24v and EV70 (CA24v-S: 5′-GTGAGTGCTTGCCCAGATTT-3′/CA24v-A: 5′-CTCCACTAGTGAGCGGTGTG-3′; and EV70-S: 5′-AGGGATTCACCAGACATTGG-3′/EV70-A: 5′-ATTTTCCACCAGGCACTCTG-3′) and nested PCR was used for the generic detection of adenoviruses [20]. The PCR products were analyzed with 1% agarose electrophoresis. All 52 swabs were cultured in fresh monolayers of HEP-2 cells. The cultures were incubated at 36°C and observed daily for a cytopathic effect. Two blind passages were performed when no cytopathic effect was observed.

### Nucleotide Sequencing and Phylogenetic Analyses

The 3Cpro and VP1 genes of the isolates were amplified from the RNAs extracted from the culture supernatants and cycle sequenced with primers 3C1/3C2 and CA24v-2407S/CA24v-3438A, respectively [6]. The products were analyzed with an ABI PRISM 3100 Genetic Analyzer (Applied Biosystems, Hitachi, Japan). The MEGA 4.0 software [21] was used for the phylogenetic analysis. The homologies among the genotypes were calculated using the Kimura two-parameter model. The phylogenetic trees were assessed using neighbor-joining cluster analysis with 1000 bootstrap replications, and only bootstrap values >70% are shown at the nodes.

### Nucleotide Sequence Accession Numbers

All sequences reported in this study were deposited in GenBank under accession number JN788169–JN788308. In total, 276 strains with complete 3Cpro sequences and 139 strains with complete VP1 sequences that were available in GenBank (isolated between 1970 and 2011) were investigated. Sixty-three 3Cpro sequences and 41 VP1 sequences, selected for their representativeness, were used to construct the phylogenetic trees. Of these strains, 38 had complete sequences for both the 3Cpro and Vp1 regions. The GenBank accession numbers of the downloaded strains are shown on the trees (**Fig. 2** and **Fig. 3**).
